# Measurement of solid food intake in *Drosophila* via consumption-excretion of a dye tracer

**DOI:** 10.1038/s41598-018-29813-9

**Published:** 2018-08-01

**Authors:** Brandon C. Shell, Rebecca E. Schmitt, Kristen M. Lee, Jacob C. Johnson, Brian Y. Chung, Scott D. Pletcher, Mike Grotewiel

**Affiliations:** 1 Department of Human and Molecular Genetics, Virginia Commonwealth University, Richmond, VA USA; 2 Human Genetics Graduate Program, Virginia Commonwealth University, Richmond, VA USA; 3 Neuroscience Graduate Program, Virginia Commonwealth University, Richmond, VA USA; 4 Department of Molecular and Integrative Physiology and Geriatrics Center, University of Michigan, Ann Arbor, Michigan, USA; 5 VCU Alcohol Research Center, Virginia Commonwealth University, Richmond, VA USA

## Abstract

Although the *Drosophila melanogaster* (fly) model is a popular platform for investigating diet-related phenomena, it can be challenging to measure the volume of agar-based food media flies consume. We addressed this challenge by developing a dye-based method called Consumption-Excretion (Con-Ex). In Con-Ex studies, flies consume solid food labeled with dye, and the volume of food consumed is reflected by the sum of the dye inside of and excreted by flies. Flies consumed-excreted measurable amounts of FD&C Blue No. 1 (Blue 1) and other dyes in Con-Ex studies, but only Blue 1 was readily detectable at concentrations that had no discernable effect on consumption-excretion. In studies with Blue 1, consumption-excretion (i) increased linearly with feeding duration out to 24 h at two different laboratory sites, (ii) was sensitive to starvation, mating status and strain, and (iii) changed in response to alteration of media composition as expected. Additionally, the volume of liquid Blue 1 consumed from capillary tubes was indistinguishable from the volume of Blue 1 excreted by flies, indicating that excreted Blue 1 reflects consumed Blue 1. Our results demonstrate that Con-Ex with Blue 1 as a food tracer is a useful method for assessing ingestion of agar-based food media in adult flies.

## Introduction

The fruit fly (*Drosophila melanogaster*) has emerged as a powerful model for investigating the effects of diet on both physiological and disease-like states. Studies in flies have revealed that diet has substantial effects on lifespan^1–5^, egg-laying^6^, metabolism^7^, fat deposition^8^, perceived nutritional value of food^9^, food choice^10^, sleep^11^ and a host of other phenotypes^4,5,12–29^. Flies are housed on solid, agar-based media for days at a time in most laboratory studies. Unfortunately, it can be challenging to determine the volume of media flies consume under routine housing conditions^30^. This lack of clarity greatly undermines the causal connections that can be drawn between diet, dietary intake and physiological measures in the fly model.

Approaches for measuring solid food consumption in flies have been described. Methods that rely solely on the accumulation of internal dye consumed from fly food can be highly problematic because the internal dye signal quickly plateaus with time^31^. Other previously described approaches record the proportion of flies extending the proboscis (mouth parts) into the food^32^ or the proportion of flies feeding on food^33^. A limitation of these two other approaches is that it is unclear if proboscis extension into food media or the proportion of flies feeding on food media equates with the volume of media consumed. Another method called FlyPAD determines feeding behavior in individual flies based on their interaction with solid food medium^34^. This highly sophisticated method quantifies a number of key parameters associated with feeding in flies, but was not designed to measure consumption of solid food media under routine laboratory conditions.

Arguably the most promising approach for measuring solid food intake in flies under typical housing conditions described to date quantitates the consumption of dietary media labeled with a radioactive tracer. Studies using this approach revealed that mated females consume more media than virgin females^35^, demonstrated^36^ or confirmed^31^ that flies adjust their intake of solid media to compensate for changes in nutrient concentrations, and that RU486 (the steroid inducer in a frequently used conditional expression strategy in flies^37,38^) reduces food consumption^23^. Unfortunately, the prospect of radioactive flies being inadvertently released from a laboratory makes this approach impractical for some users. Additionally, the radioactive chemicals (e.g. leucine, ATP, CTP, etc.^31,35,36^) used in this approach must be metabolized and then incorporated into long-lived molecules within the fly to be detected. The requirement for metabolism and macromolecular incorporation, two processes that might change in response to altered dietary composition, is a potential confound for using radioactive tracers to measure ingestion of food media.

Here, we report the development of a dye-based method called Consumption-Excretion (Con-Ex) for assessing solid food intake in flies. Flies in Con-Ex studies consume solid media labeled with FD&C Blue No. 1 for hours to days at a time. The amount of food media consumed is reflected by the sum of the dye inside flies and the dye excreted from flies. Our studies show that Con-Ex is suitable for detecting the effects of starvation, fly strain, mating status and changes in the composition of the diet on media intake. Additionally, our studies show that Con-Ex has utility in multiple laboratory environments. Con-Ex is technically straightforward, inexpensive, and requires equipment found in virtually any laboratory. The Con-Ex method should be suitable for assessing consumption of solid food media in flies in a wide range of laboratories, especially those in which the use of radioactive food labels might be challenging.

## Results

**Figure 1:**
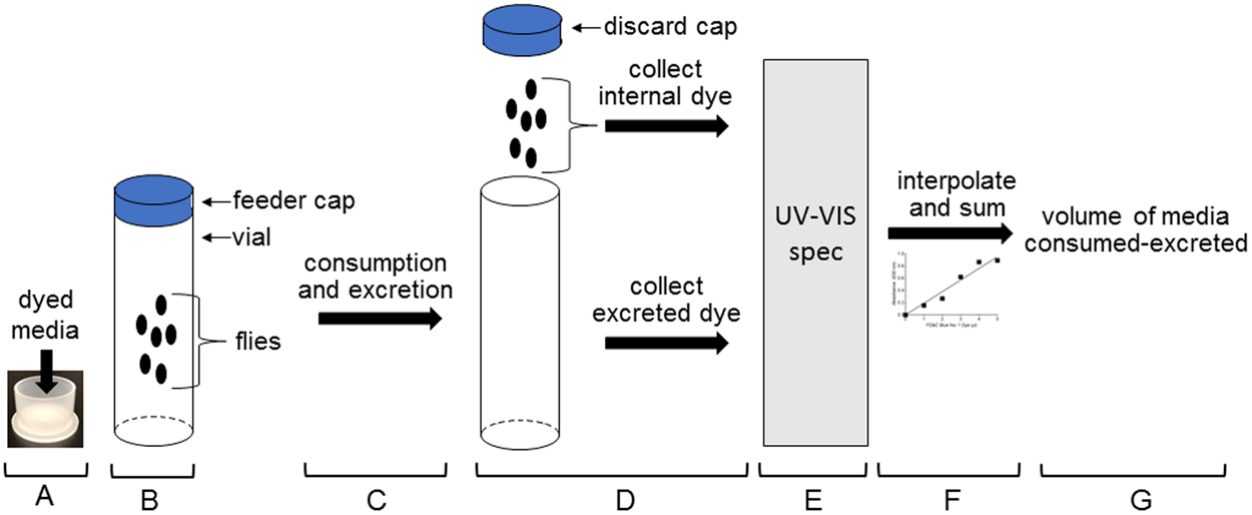
Schematic of the Con-Ex method in flies. See main text for details.

The goal of these studies was to develop and validate a simple, reliable, inexpensive method for measuring consumption of solid food medium in *Drosophila*. Several related ideas influenced the method we developed: (1) many inexpensive food dyes are commercially available; (2) when flies consume food medium labeled with dye, accumulation of dye inside flies plateaus over time because dye intake is quickly balanced by excretion of the dye as waste^31^; (3) dye in excreted waste should accumulate over time within vials in which flies are housed; and (4) at the conclusion of a feeding experiment, the sum of the dye found inside flies and the dye excreted from flies should reflect food intake. Considering these ideas, we developed the Consumption-Excretion (Con-Ex) method. Major experimental steps in Con-Ex studies include (i) placing adult flies in empty food vials containing a single removable feeder cap that contains solidified food medium labeled with dye (Fig. 1A,B); (ii) allowing flies to consume food from the feeder caps and excrete waste in the vials for prescribed periods of time (Fig. 1C); (iii) at the conclusion of food exposure, extracting the dye inside flies (Internal dye, INT) and the excreted dye (ExVial, ExMedium) (Fig. 1D); (iv) quantitating the dye in these extracts via spectrophotometry (Fig. 1E); (v) converting the dye absorbance to volume via interpolation from standard curves of pure dye (Fig. 1F); and (vi) calculating the consumed-excreted volume of media (Fig. 1G). See Table S1 for definitions of all measures.

We initiated our Con-Ex studies by identifying a suitable dye (and its concentration) and then validated the method in a number of ways including (i) determining whether it could detect the effects of feeding duration, genetic background, starvation and media composition on food intake, (ii) exploring whether the method works in multiple laboratories, and (iii) determining whether consumed dye equated with excreted dye.

### Identification of dyes and appropriate fly density for Con-Ex

**Figure 2:**
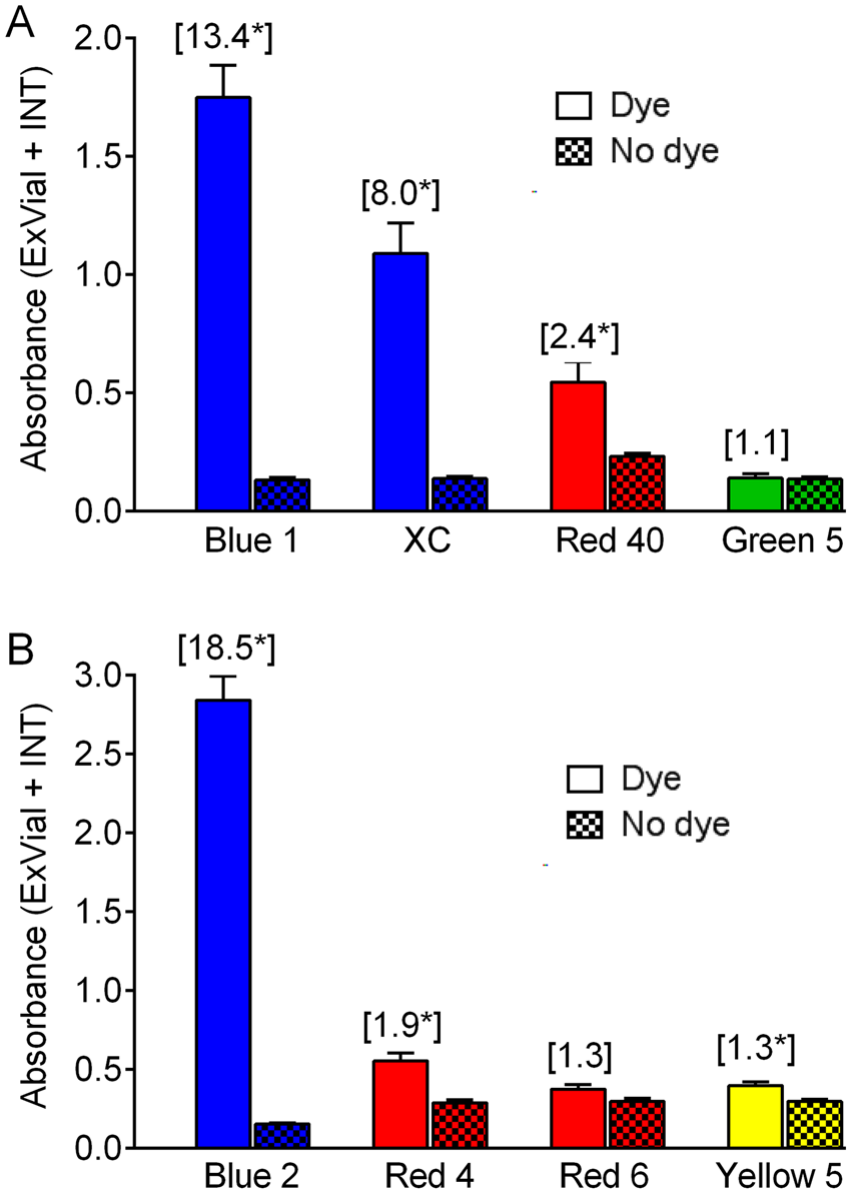
Signal-to-noise for candidate food dyes in Con-Ex. Data are the summed absorbance values of INT + ExVial. Dye (simple filled bars): absorbance values from flies fed media containing 1% of the indicated dyes (signal). No Dye (hatched bars): absorbance values from flies fed media without dye (noise). Control r[A] female flies (20/vial) consumed media and excreted waste products in vials for 24 h. Numbers in brackets indicate signal-to-noise ratios. Signal (Dye) was greater than noise (No dye) in experiments with (panel A) Blue 1, xylene cyanol (XC) and Red 40, and (panel B) Blue 2, Red 4 and Yellow 5 (*individual two-tailed t tests, p = 0.007 to <0.0001, n = 5–6).

To identify dyes suitable for Con-Ex studies, we provided flies (r[A], a standard control strain in the Grotewiel laboratory) with food containing 1% w/v of several different dyes for 24 h and then assessed the signal to noise (i.e. absorbance of ExVial + INT dye divided by the background absorbance). The dyes FD&C Blue No. 1, xylene cyanol and FD&C Red 40 (Fig. 2A), as well as the dyes FD&C Blue No. 2, FD&C Red 4 and FD&C Yellow 5 (Fig. 2B) all had significantly greater signal than noise. Based on their large signal to noise ratios and the absolute magnitudes of their signals, we chose to further explore the utility of FD&C Blue No. 1 (Blue 1), xylene cyanol (XC) and FD&C Blue No. 2 (Blue 2) in Con-Ex studies. The other dyes tested (Red 40, Green 5, Red 4, Red 6 and Yellow 5) might have had smaller signals in Con-Ex studies because they are aversive tastants, are metabolized after being consumed, have pharmacological properties that suppress medium consumption or excretion, or other reasons and were not considered further.

**Figure 3:**
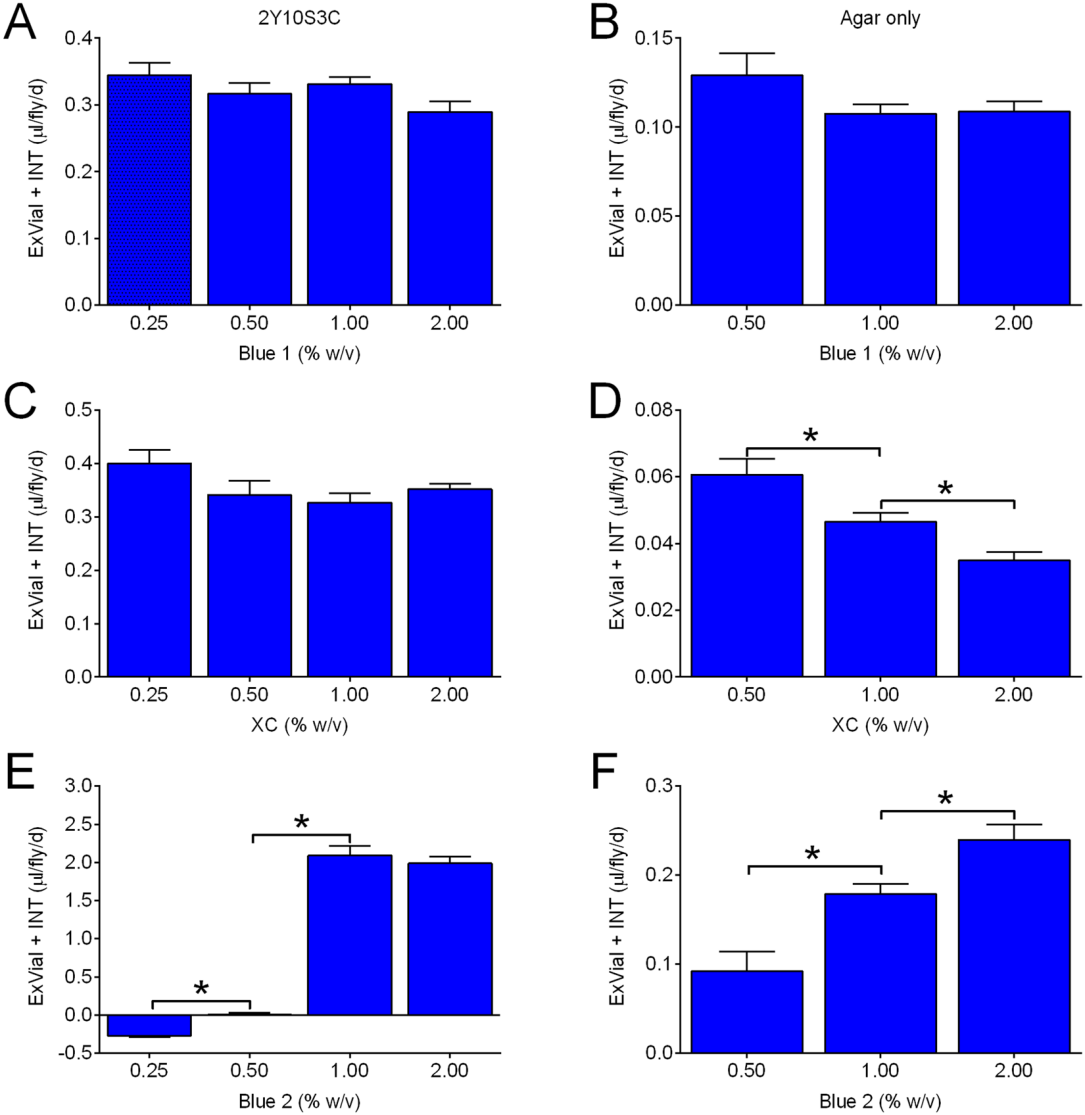
Effect of dye concentration on Con-Ex. Control r[A] females consumed 2Y10S3C (**A**,**C**,**E**) or media containing only 1% agar (**B**,**D**,**F**) with the indicated concentrations of Blue 1 (**A**,**B**), xylene cyanol (XC, panels C,D), or Blue 2 (**E**,**F**). Flies consumed media and excreted waste in the vials for 24 h. ExVial + INT dye was not affected by the concentration of Blue 1 in 2Y10S3C (**A**) or agar-only media (**B**) (individual one-way ANOVAs; 2Y10S3C, p = 0.2255; agar-only medium, p = 0.1533; n = 8–20 in panel A and 8 in panel B). ExVial + INT dye was not affected by the concentration of xylene cyanol in 2Y10S3C (**C**, one-way ANOVA, p = 0.0839, n = 16), but increasing concentrations of xylene cyanol in agar-only media decreased ExVial + INT dye (**D**; one-way ANOVA, p < 0.0001, n = 8). The concentration of Blue 2 in 2Y10S3C (**E**, one-way ANOVA, p < 0.0001, n = 16) and agar-only media (**F**, one-way ANOVA, p < 0.0001, n = 16) significantly increased ExVial + INT dye. Asterisks indicate significantly different pair-wise groups in **D**,**E** and **F** (*Bonferroni’s multiple comparisons, p = 0.0493 to <0.0001).

Ideally, the food tracers used in a feeding method should not significantly influence consumption of the media being consumed. To determine if Blue 1, XC or Blue 2 satisfied this criterion, we fed flies increasing concentrations of the dyes in either our standard fly medium (2% yeast, 10% sucrose, 3.3% cornmeal, 1% agar and antimicrobials (hereafter 2Y10S3C), Fig. 3A,C,E) or in a medium containing 1% agar only (Fig. 3B,D,F). We reasoned that using two different media might reveal food × dye interactions (i.e. food-dependent effects of dye). Note that unless indicated otherwise we hereafter report the volume of dyed media consumed determined from interpolation of standard curves of pure dye.

The concentration of Blue 1 (0.25–2% w/v) in either 2Y10S3C (Fig. 3A) or agar-only media (Fig. 3B) had no effect on the sum of INT and ExVial dye, consistent with a previous report showing that Blue 1 does not impact consumption^31^. The dose-effects of the other dyes were more complex. Consumption-excretion of 2Y10S3C was not affected by 0.25–2.0% XC (Fig. 3C), but this dye dose-dependently decreased consumption-excretion of media made with agar only (Fig. 3D). In contrast, increasing concentrations of Blue 2 increased consumption-excretion of both 2Y10S3C (Fig. 3E) and agar-only media (Fig. 3F). Since changes in consumption-excretion driven by food tracers such as XC and Blue 2 could be a significant experimental confound, we focused all subsequent studies on Blue 1.

To identify the appropriate density of animals to use in Con-Ex studies, we varied the number of flies/vial during 24 h feeding experiments. ExVial + INT measured with Blue 1 was comparable when 10–20 flies per vial were used, but it decreased significantly with 30 flies per vial (Fig. S1). Although we do not understand why consumption-excretion per fly decreased as the density of animals increased, these results indicate that using a consistent number of flies, and 20 or fewer flies, is an important practical consideration in Con-Ex studies with Blue 1 as a tracer under the conditions used in these experiments.

### Con-Ex time-course studies under different laboratory conditions

**Figure 4:**
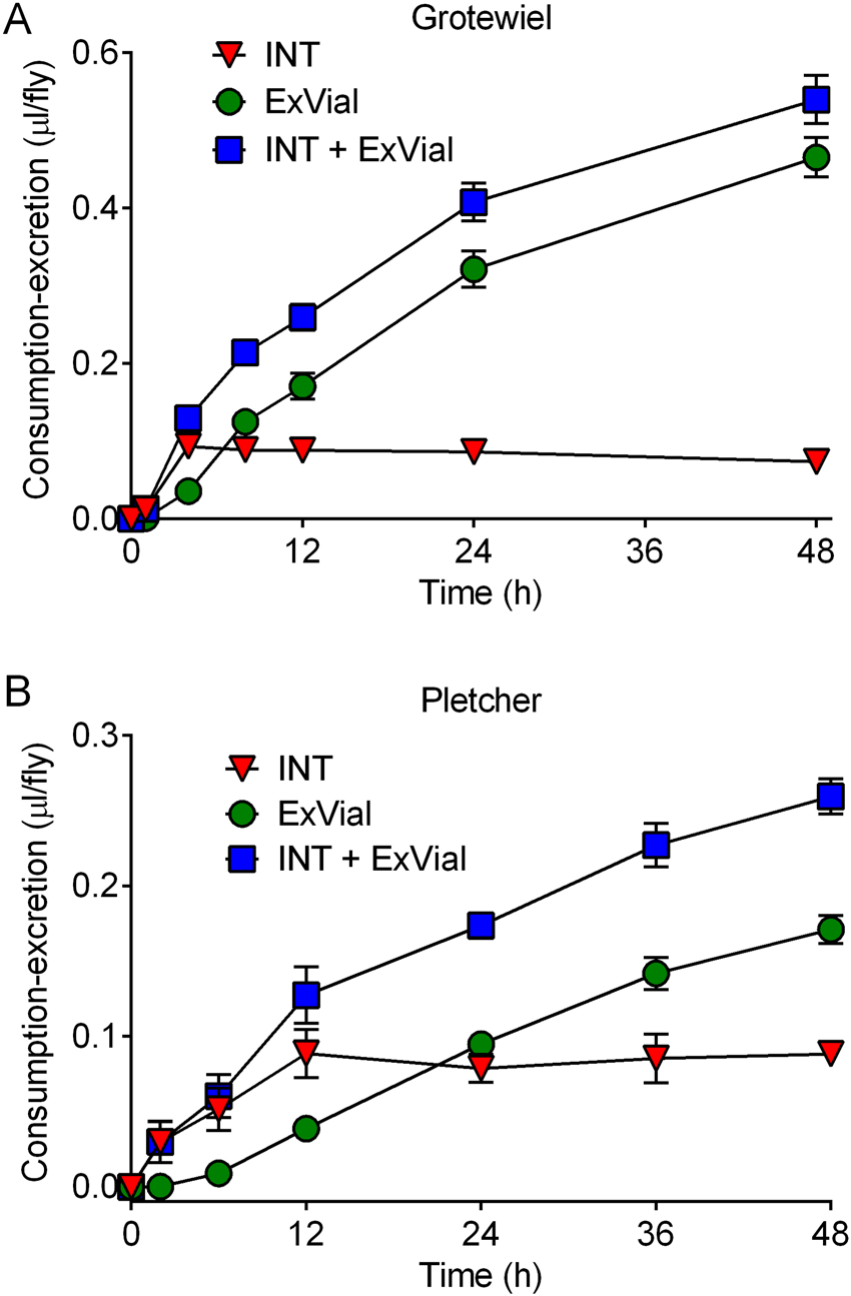
Con-Ex time-courses. Control r[A] (**A**) and Canton-S (**B**) females consumed medium labeled with 1% Blue 1 and excreted waste for the indicated times (X-axes). Data are INT, ExVial and ExVial + INT from the Grotewiel (**A**) and Pletcher (**B**) laboratories. Time influenced INT and ExVial (one-way ANOVAs; panel A, p < 0.0001; panel B, p < 0.0001; n = 8 at each time-point).

The data in Figs 2, 3 and S1 are from experiments in which flies consumed-excreted media for 24 h. To address how the duration of Con-Ex studies influenced the results, we performed time-course experiments with Blue 1 as a food label using r[A] (Fig. 4A) and Canton-S (Fig. 4B) females. Importantly, the data in Fig. 4A,B were generated in the Grotewiel and Pletcher laboratories, respectively. In both studies, the amount of INT Blue 1 plateaued or peaked by 4 (Fig. 4A) or 12 (Fig. 4B) hours after the initiation of feeding. There was an initial lag in appearance of ExVial Blue 1 (Fig. S2A,B), but thereafter the amount of ExVial dye accumulated with time (Fig. 4). ExVial and ExVial + INT dye accumulation approximated linear functions in both r[A] and Canton-S females (linear regression; R^2^ = 0.8330 to 0.9387; p < 0.0001 in all cases). The quantitative differences between the results in Fig. 4A,B are presumably driven by differences in environment including food media, strain and age of the animals (see Methods). Our results with different strains and ages of flies reared under different conditions suggest that accumulation of consumed-excreted Blue 1 can be measured out to 24 or possibly 48 h without major concerns regarding ceiling effects, that consumption of Blue 1 precedes its excretion, and that the Con-Ex method works well in different laboratories.

### Detecting strain, starvation, mating status and media composition effects

**Figure 5:**
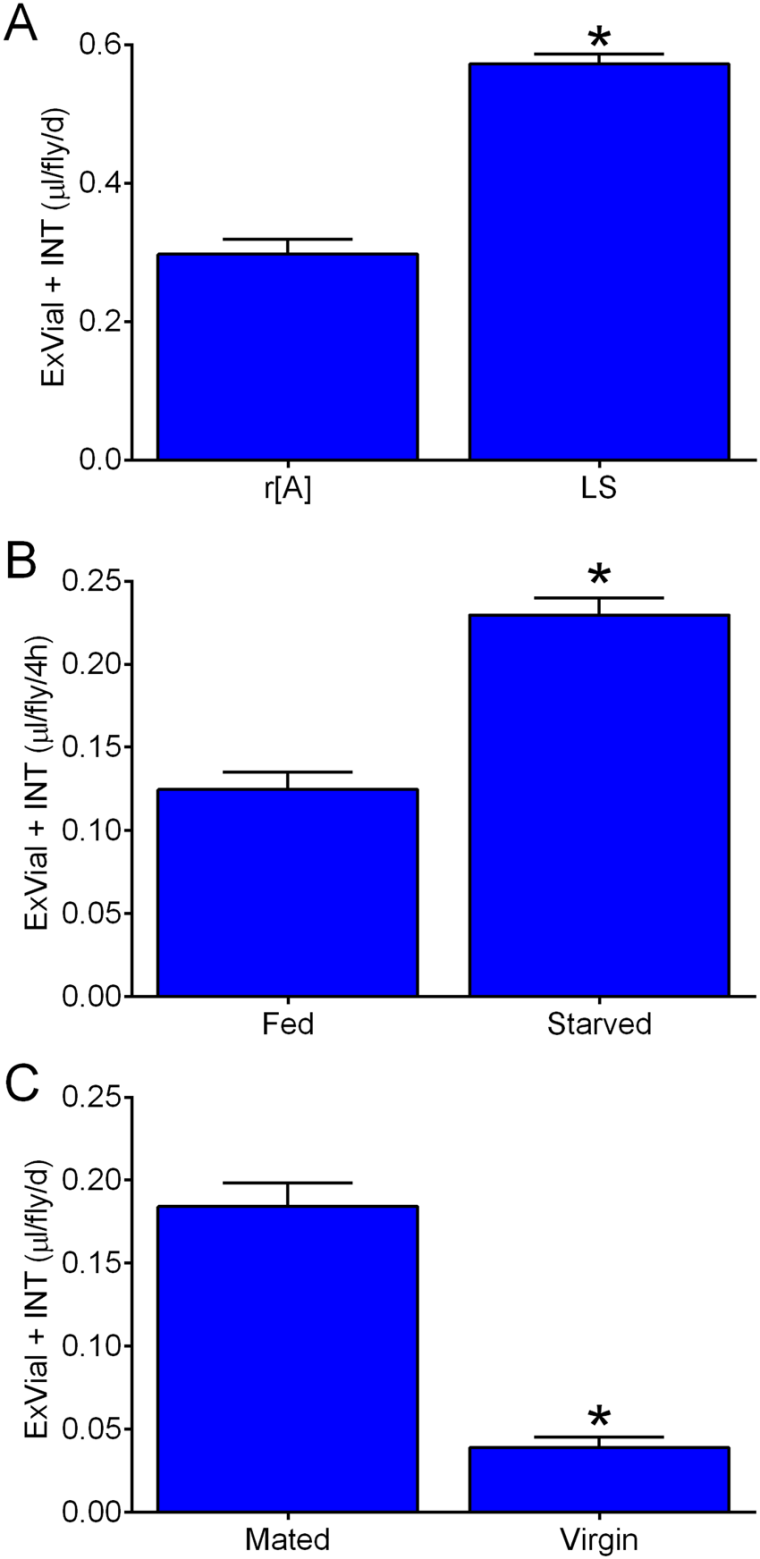
Genetic background, starvation and mating influence Con-Ex. Control females were fed 2Y10S3C containing 1% Blue 1 for 24 h (**A**,**C**) or refed this medium for 4 h (**B**). ExVial + INT was significantly greater in LS compared to r[A] (**A**; *two-tailed t test, p < 0.0001, n = 8), was greater in starved compared to fully fed r[A] females (**B**; *two-tailed t test, p < 0.0001, n = 8), and was greater in mated compared to virgin r[A] females (**C**; *two-tailed t test, p < 0.0001, n = 10).

To address the ability of the Con-Ex method to detect differences across fly strains, we assessed 24 h consumption-excretion in control r[A] and control Lausanne-S (LS) females fed 2Y10S3C medium. ExVial + INT in LS flies was ~twice that of r[A] (Fig. 5A), indicating that the Con-Ex method can readily detect the effects of strain (genetic background in this case) on consumption of food media. There was no difference in total body weight of r[A] and LS females (0.939 ± 0.004 and 0.991 ± 0.026 mg, respectively; t test, p = 0.076; n = 8) and therefore the differences in Con-Ex between these two strains in unrelated to body size. Although the effects of genetic background on *Drosophila* feeding behavior are widely appreciated (e.g.^31^), the difference in Con-Ex between the r[A] and LS strains we report is novel.

A period of starvation in flies increases their consumption of liquid food media^10,11,39,40^. To determine whether starvation increases consumption of solid media, we compared 4 h consumption-excretion in fully fed and starved flies. We assessed consumption-excretion at 4 h (as opposed to a more typical 24 h) in these studies because the effects of starvation might wane once flies return to a fully fed state. Prior starvation substantially increased consumption-excretion of media labeled with Blue 1 in r[A] females (Fig. 5B). Con-Ex studies are therefore capable of detecting the consequences of short-term starvation on refeeding in flies.

Mated females consume more solid food media than virgin females when consumption is measured by a radioactive tracer^31^. To assess the utility of Con-Ex for detecting the effect of mating status on medium consumption, we compared 24 h consumption-excretion in mated and virgin females. Mated females consumed-excreted more medium than virgin females (Fig. 5C) in Con-Ex as expected^31^.

**Figure 6:**
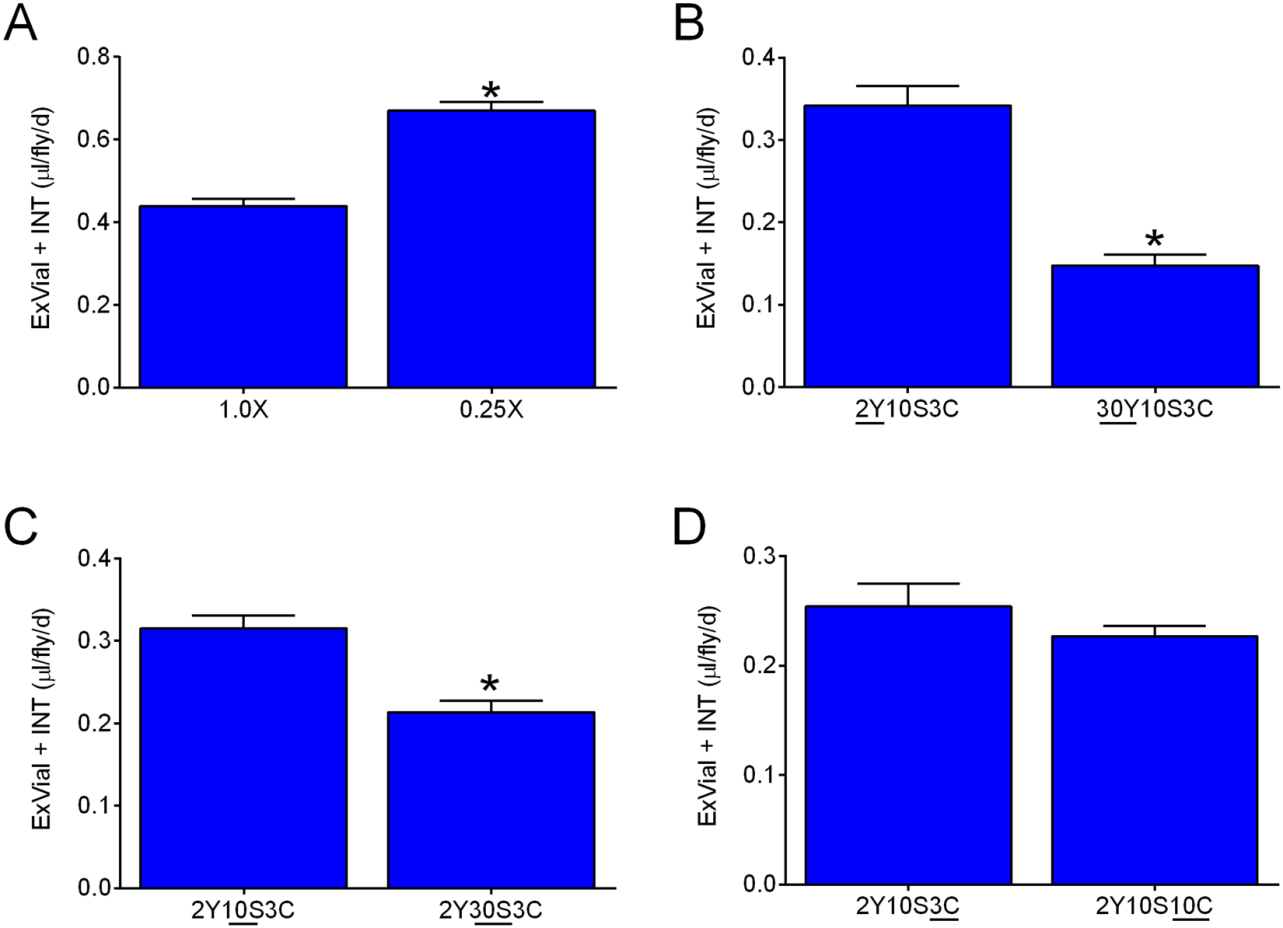
Composition of food media influences Con-Ex. Control r[A] females consumed the indicated media labeled with 1% Blue 1 and excreted waste for 24 h. ExVial + INT was greater in flies that consumed 0.25X medium than in flies fed 1.0 × 2Y10S3C medium (panel A, *two-tailed t test, p < 0.0001, n = 16), in flies fed medium containing 2% yeast (2Y10S3C) compared to 30% yeast (30Y10S3C) (panel B, *two-tailed t test, p < 0.0001, n = 8) and in flies fed medium containing 10% sucrose (2Y10S3C) compared to 30% sucrose (2Y30S3C) (panel C, *two-tailed t test, p = 0.0003, n = 8). (**D**) ExVial + INT was indistinguishable in flies fed medium containing 3% cornmeal (2Y10S3C) and 10% cornmeal (2Y10S10C) (two-tailed t test, p = 0.2532, n = 8).

Flies exhibit compensatory feeding (i.e. an increase in the volume medium consumed as the concentration of nutrients is decreased^31,36^). As expected, decreasing the concentration of all components of 2Y10S3C (1.0X) medium to 0.25X led to an increase in ExVial + INT (Fig. 6A). Furthermore, increasing the concentration of yeast from 2% to 30% or increasing the sucrose concentration from 10% to 30% significantly reduced ExVial + INT (Fig. 6B,C, respectively). Increasing the concentration of corn meal from our standard 3.3% to 10% (the practical maximum), however, did not significantly alter ExVial + INT (Fig. 6D). Taken together, the data in Fig. 6 indicate that Con-Ex studies with Blue 1 can detect the expected changes in feeding behavior in response to alteration of food media composition. Our results also suggest that flies might alter their dietary consumption in response to changes in some (yeast and sucrose), but not all (cornmeal), food components.

### Coupling of CAFE and Con-Ex to assess Blue 1 as a tracer for consumption

**Figure 7:**
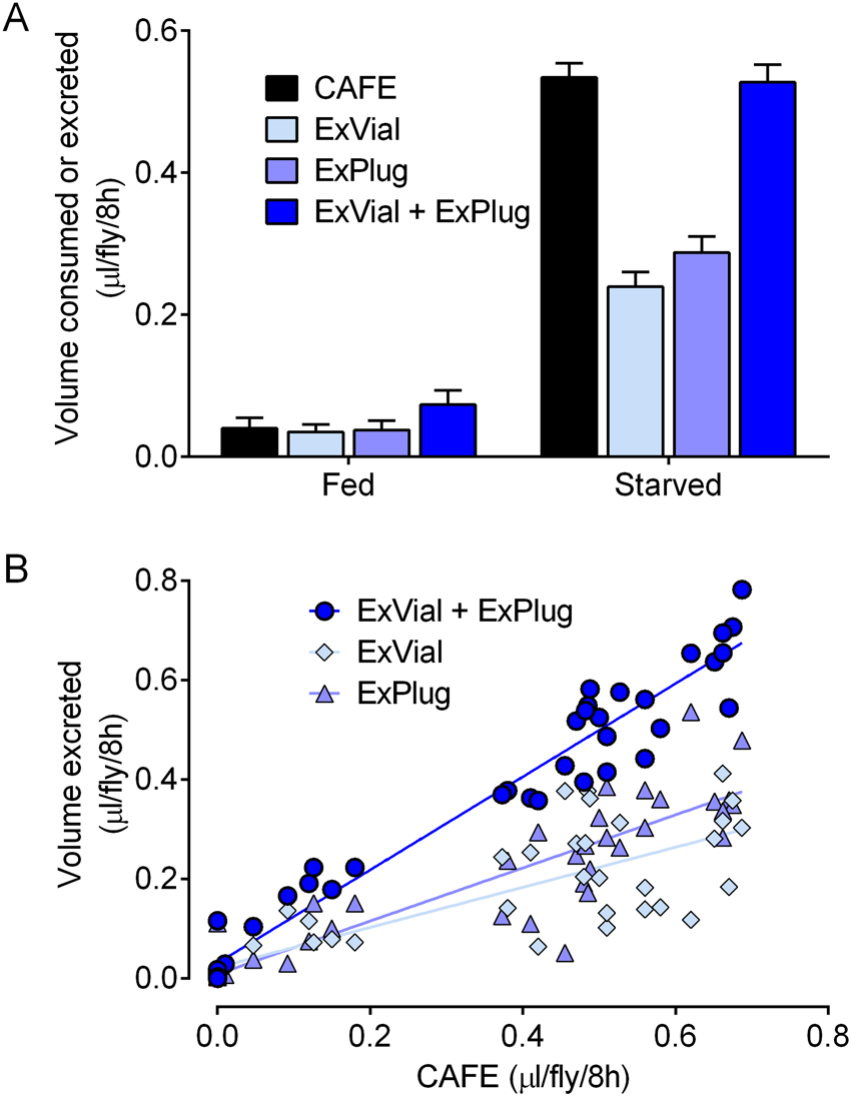
CAFE consumption of Blue 1 equates with excretion of Blue 1. (**A**) In control r[A] females, the volume of liquid medium consumed from capillary feeders (CAFE) and excreted (ExVial + ExPlug) was significantly increased by starvation, but the two measures were statistically indistinguishable (two-way ANOVA; starvation, p < 0.0001; measure, p = 0.5302, interaction, p = 0.3537; n = 18–24 per group). (**B**) Additional analysis of the data in panel A. The volume of liquid medium consumed from capillary feeders (CAFE) correlated with ExVial, ExPlug and ExVial + ExPlug (Pearson correlations: R^2^ = 0.6573, R^2^ = 0.8165, R^2^ = 0.9539, p < 0.0001 for all, n = 42). Lines are best-fit linear regressions.

The capillary feeding (CAFE) assay has been used extensively to measure consumption of liquid diets in flies^10,11,31,39,41^. We coupled CAFE and Con-Ex methods in a single experimental design to address whether the volume of Blue 1 excreted might reflect the amount of Blue 1 consumed. In pilot studies, we found that flies consuming liquid media (labeled with Blue 1) from capillary tubes excreted on both the vial wall and the foam plug holding the capillary tubes (not shown, but see below). Importantly, Blue 1 applied directly to foam plugs can be collected and quantitated (Fig. S3A), thereby allowing it to be measured as part of the excreted dye signal. Additionally, while flies fed medium containing Blue 1 have readily detectable INT dye after 24 h of access to the medium, flies contain no detectable INT dye 24 h after being switched to medium without dye (Fig. S3A,B). We therefore performed CAFE-excretion studies in which flies were provided access to liquid 5% sucrose media containing Blue 1 in capillary tubes for 8 h (to measure the amount of labeled media they consumed and also allow excreted dye to begin accumulating) and then were switched to capillary tubes containing liquid medium without dye for 24 h (to allow INT dye from prior consumption to be excreted). We used both fed and starved flies to generate a range of liquid medium consumption and excretion in these experiments in anticipation of correlation analyses (Fig. 7B).

Previously fed flies consumed little liquid medium from capillary tubes while starved flies consumed considerably more as expected given the relatively short feeding period employed (Fig. 7A). Flies excreted a substantial amount of dye on the vial walls (ExVial) and on the foam plugs (ExPlug) (Fig. 7A). The amount of Blue 1-labeled liquid medium consumed by flies (CAFE) was indistinguishable from the amount of Blue 1 excreted (ExVial + ExPlug) in both fed and starved flies (Fig. 7A). Additionally, the amount of liquid Blue 1 consumed from capillary tubes (CAFE) correlated with (i) ExVial + ExPlug, (ii) ExVial and (iii) ExPlug (Fig. 7B). The volume of Blue 1 excreted therefore reflects, and is potentially equivalent to, the volume of Blue 1 consumed by flies under the conditions used in these studies.

### Con-Ex in males

The results reported in Figs 2–7 and S1–S3 are from studies using females. We therefore performed an additional set of experiments to address whether Con-Ex is similarly suitable for studies in males. INT dye plateaued quickly and ExVial as well as ExVial + INT accumulated in a largely linear fashion out to ~48 h in both in r[A] and Canton-S males (Fig. S4A, Grotewiel lab; Fig. S4B, Pletcher lab). Additionally, ExVial + INT was greater in LS compared to r[A] males (Fig. S4C) and starvation increased ExVial + INT in r[A] males (Fig. S4D). The results from males and females are qualitatively similar overall, indicating that Con-Ex is suitable for studies in both sexes.

### Excretion of dye on the food medium

In addition to INT and ExVial dye, we anticipated that flies in Con-Ex studies would excrete dye on the food medium (excreted on medium, ExMedium). To address this possibility, we fed control flies 2Y10S3C food labeled with Blue 1 for 4 h (vial 1) and transferred the dye-fed flies to new vials containing feeder caps with medium without dye (vial 2). Flies then excreted the previously consumed dyed media for 24 h in vial 2 and we collected the resulting ExVial and ExMedium dye. Importantly, flies excreted the vast majority (if not all) of the Blue 1-labeled contents of their gastrointestinal tract in 24 h (Fig. S3B) and Blue 1 added to the surface of the food medium in feeder caps was readily recovered by water extraction (Fig. S3C).

**Figure 8:**
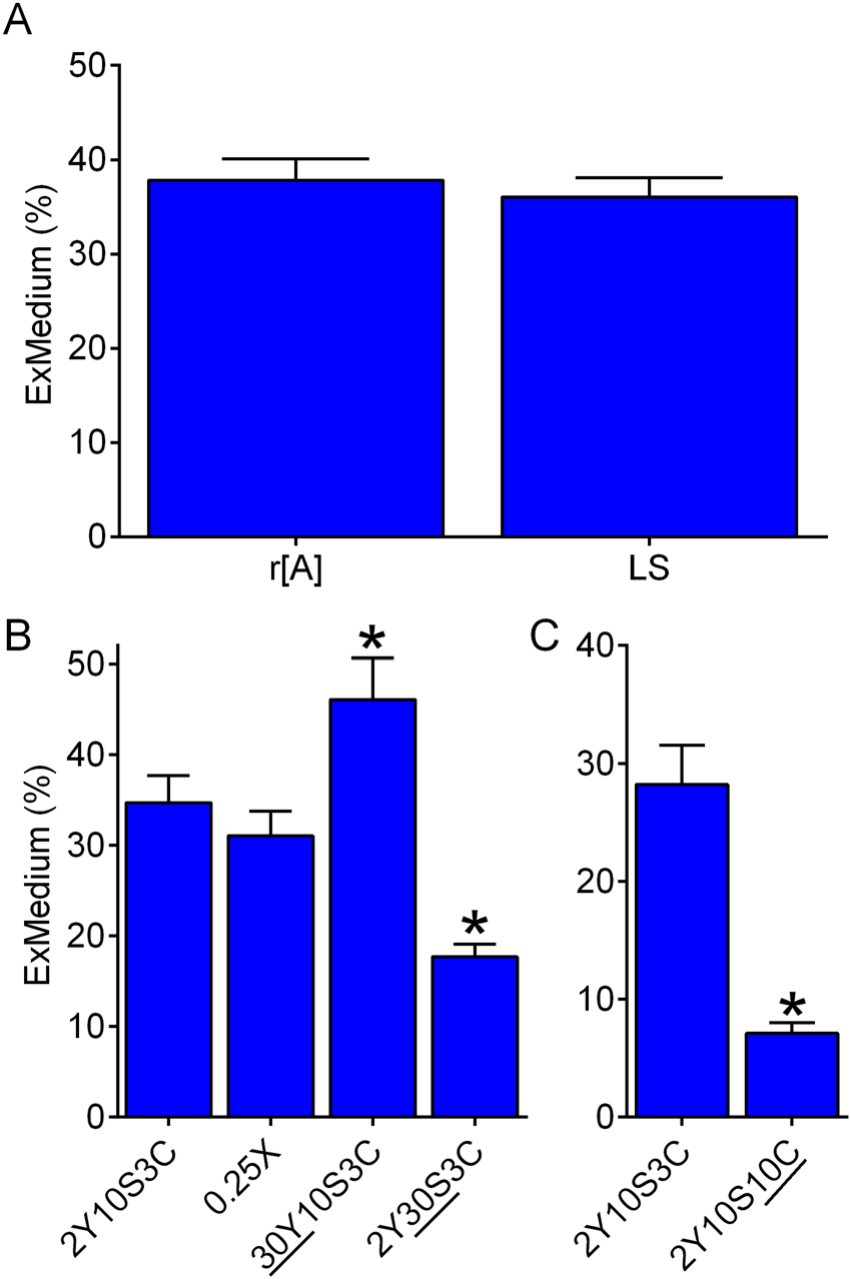
Excretion on the food medium in Con-Ex. Data are percentage of excreted dye found on the food medium. (**A**) The percentage of dye on 2Y10S3C medium (ExMedium %) was indistinguishable in r[A] and LS females (two-tailed t test, p = 0.5738, n = 8). (**B**) Food medium composition influenced ExMedium % (one-way ANOVA, p < 0.0001, n = 6–10). Compared to 2Y10S3C, ExMedium % was significantly greater on 30Y10S3C (*Bonferroni’s multiple comparison, p = 0.0366) and lower on 2Y30S3C (*Bonferroni’s multiple comparison, p = 0.0009. (**C**) ExMedium % was lower on 2Y10S10C than on 2Y10S3C (*two-tailed t test, p < 0.0001, n = 8).

When provided with 2Y10S3C medium, control r[A] and LS females excreted comparable percentages of waste dye on the food medium (ExMedium %, Fig. 8A). Interestingly, whereas diluting 2Y10S3C medium to 0.25X had no effect on ExMedium %, increasing the yeast concentration to 30% led to an increase in ExMedium % (Fig. 8B). Additionally, increasing the sucrose concentration to 30% and increasing the cornmeal concentration to 10% both decreased ExMedium % (Fig. 8B,C). The simplest interpretation of these data is that ExMedium % might not vary substantially across control strains fed the same diet, but it is influenced by media composition.

The most comprehensive approach when using Con-Ex would in principle be to measure INT, ExVial and ExMedium simultaneously. This is unfortunately impossible when using a single food tracer because the medium must be labeled to track it, the flies must have physical access to the medium to consume it and access to the medium allows flies to deposit waste products on the already labeled medium. Given this experimental limitation, we explored whether including ExMedium measurements was necessary to detect changes in consumption-excretion by reanalyzing the data in Figs 3A, 4A, 5A, 6A–D and S1 (data from experiments in which the relevant measurements were made). In these analyses, we calculated the adjusted volume excreted in the vial (ExAdj) as ExVial × [1/(1 − fraction ExMedium)] (the final bracketed term accounts for the fraction of dye on feeder caps). In addition to ExMedium, we also explored whether including INT had a substantial impact on Con-Ex data interpretation.

Using the results of 8 independent studies shown in Figs 3A, 4A, 5A, 6A–D and S1 we performed a total of 19 statistical tests on the four different measures: ExVial alone, ExAdj alone, ExVial + INT and ExAdj + INT (Table S3). Note that due to low dye signal from groups with low dye concentrations (Fig. 3A), lower number of flies (Fig. S1) and after shorter periods of feeding (Fig. 4A), determining ExMedium in these studies was not possible and consequently we assumed that ExMedium (and therefore the calculation of ExAdj) was not affected by dye concentration, the number of flies per vial or time on the medium. In 17 of 19 tests, the statistical outcomes were indistinguishable across all four measures (Table S3). Of the two cases in which the statistical tests were not consistent across the four measures, one involved the percent change at early time-point in time-course studies during which INT is a substantial fraction of the total dye measured (Table S3, Fig. 4A, Bonferroni’s: 1–4 h) and the other involved a food medium with the highest practical concentration of cornmeal which influences ExMedium and therefore ExAdj (Table S3, Fig. 6D, two-tailed t test). Importantly, the percent changes in ExVial, ExAdj, ExVial + INT and ExAdj + INT were highly correlated, comparable in size, and in the same direction in virtually all cases (Table S3). Our reanalysis suggests that INT and ExMedium do not contribute significantly to interpretations in many cases and therefore that measuring ExVial alone might be sufficient to detect major changes in Con-Ex in most studies.

### Con-Ex power analyses

An important consideration regarding the utility of any experimental method is the number of replicates that must be performed to detect differences of varying magnitude (i.e. power). Using the average standard deviations for ExVial, ExAdj, ExVial + INT and ExAdj + INT derived from 23 experiments with control r[A] females, we found that all four measures had similar power to detect differences between groups (Fig. S5), consistent with the statistical reanalysis of these four measures (Table S3). Additionally, we found that 30%, 20% and 10% differences between two mean values can be detected at 80% power in Con-Ex studies with 4–6, 8–11 and 30–50 replicates, respectively (Fig. S5). Detection of differences of 20% or greater in Con-Ex studies therefore requires a reasonable number of replicates to be performed. The number of replicates required to detect differences in Con-Ex appears comparable to CAFE, but greater than when using radioactive methods^31^.

## Discussion

Despite the growing interest in studies on diet in flies, it is often very challenging to determine the amount of dietary media consumed when flies are housed on solid, agar-based food^30^. We developed Con-Ex as a dye-based method for determining intake of solid media in *Drosophila*. Flies are provided continuous access to agar-based media for hours to days at a time in Con-Ex studies, mimicking routine fly housing conditions. Flies in Con-Ex studies consume food media labeled with dye and then excrete dyed waste throughout their environment within the vial for the duration of the experiment. Of the dyes that we tested in Con-Ex experiments, FD&C Blue No. 1 is the only dye that has a strong absorbance signal, has reasonably low background noise, and does not influence media consumption. Con-Ex studies with Blue 1 detect the predicted influences of feeding duration, strain, starvation, mating status and food composition on media ingestion. Additionally, the volume of Blue 1 consumed in liquid medium is comparable to, if not identical to, the volume of excreted Blue 1. Furthermore, Con-Ex data generated in the Grotewiel and Pletcher laboratories were qualitatively similar. Our studies indicate that Con-Ex with Blue 1 as a food tracer is useful for assessing solid media consumption in adult flies. Identifying additional food dyes, particularly dyes that have distinct detection requirements (i.e. dyes with absorbances that do not interfere with the detection of Blue 1) would be valuable, for example, in studies aimed at measuring concurrent consumption-excretion of different media, experiments that require a specific absorbance spectrum due to interference by a transgenic marker, etc. Labeling food with fluorescent tracers like rhodamine B or fluorescein^39,42^ might increase the sensitivity of or otherwise further broaden the utility of the Con-Ex method.

The accumulation of ExVial or ExVial + INT dye rises in a largely linear fashion out to ~24 h in Con-Ex studies, but thereafter it appears to begin to plateau as described previously in studies using a radioactive tracer^31^. Since flies are exposed to and consume the same food medium over several days in Con-Ex and radioactive labeling studies (as well as under standard housing conditions), non-linear food intake after ~24 h on the medium might be the norm with routine fly husbandry. Additional studies are needed to determine whether the non-linear consumption at later feeding time-points is related to time-dependent loss of water from the medium, accumulation of waste on the medium, or other changes.

The dye used to label food media in Con-Ex studies as described here can be detected in 3 samples: INT, ExVial, and ExMedium. In studies that included all relevant measures, we calculated the total amount of excreted dye (ExAdj) by adjusting ExVial to account for the volume excreted on the food medium. Although ExAdj + INT is arguably the most comprehensive value to assess in Con-Ex studies because it likely reflects the total volume of media consumed, we found that assessing ExVial alone was typically sufficient. In the majority of studies, statistical analyses with or without ExMedium or INT led to the same interpretations and the magnitudes as well as the directions of change in the volumes consumed-excreted were comparable whether ExMedium or INT were considered. Thus, manipulations that cause large effects could likely be identified—at least in a preliminary fashion—by measuring ExVial alone.

Flies stand on or walk across the surface of the food medium in Con-Ex studies. This physical interaction with the food medium, which is required for consumption, raised the possibility that some of the dye we measured as excreted might instead have been transferred onto the vial wall by the flies’ appendages, thereby potentially confounding the use of Con-Ex to measure consumption per se. Several observations lead us to believe that the vast majority of dye that is deposited in the vial during Con-Ex studies is from consumption and subsequent excretion of the food medium. In our time-course studies with Blue 1, we detect greater amounts of INT dye than ExVial dye at early time-points and there is a lag of at least 1 h before ExVial dye can be detected. This strongly suggests that flies can interact with the medium to consume it without transferring detectable amounts of dye from the food to the vial on their appendages and is wholly consistent with dye consumption preceding dye excretion. Additionally, in Con-Ex studies dye is clearly visible inside of flies and in excretion products on the walls of vials, and the total volume of media consumed is comparable to previously reported volumes determined using radioactive food labels^31^. The most parsimonious interpretation of our data is that the vast majority of the detectable dye in Con-Ex experiments is derived from consumed food media.

One of the most widely used approaches for measuring solid media consumption in flies is based on the internal accumulation of a radioactive tracer^31,35,36^. Consumption of media labeled with some radioactive tracers leads to the progressive accumulation of the tracer within flies for up to 3 days^31^. Although the utility of this method is well established, the possibility of inadvertently releasing radioactive flies in a laboratory could make this approach challenging to implement in some settings. Additionally, the radioactive chemicals (leucine, ATP, CTP, etc.^31,35,36^) in this approach must be metabolized and then incorporated into long-lived molecules within the fly to be detected. This requirement for macromolecular incorporation is a potential confound for using accumulation of radioactive tracers given that changes in absorption of nutrients from the gut or metabolism of the tracers could occur in response to dietary manipulations.

FlyPAD is another method for assessing feeding behavior in *Drosophila* fed solid food media^34^. FlyPAD is a highly sophisticated approach that measures the interactions of individual flies with solid food medium over relatively short time periods of up to ~1 h. Major strengths of the FlyPAD method include that it assesses behavior in individual flies, it captures very detailed information regarding feeding behavior, and the number and duration of fly interactions with the food medium correlate with estimates of medium consumption^34^. In contrast to FlyPAD, Con-Ex requires equipment found in virtually any modern laboratory, was developed primarily to estimate consumption of solid medium, and assesses consumption of medium under routine housing conditions in groups of animals on media for hours to days. Although both FlyPAD and Con-Ex could certainly be adapted for uses beyond those described here, the two methods are quite different technically and are suitable for addressing distinct questions related to the biology of feeding in flies.

Of the previously described methods, Con-Ex is most similar to those based on the accumulation of radioactive isotopes. Both Con-Ex and the radioactivity accumulation methods label food with a tracer that does not impact consumption, report essentially linear rises in the volume of media consumed out to 24 h or more, can be used for feeding durations lasting hours to days under conditions mimicking routine housing conditions, detect the effects of genetic background, starvation and mating status on consumption, and are able to measure the consequences of changing the nutritional composition of fly media^31^. Advantages of the radioactivity-based methods are that they are operationally straightforward and sensitive. Advantages of the Con-Ex method are that it uses a readily available dye tracer, does not rely on radioactivity, is inexpensive and circumvents potential confounds associated with metabolism of the food label. Our results using Con-Ex with Blue 1 indicate that it is a suitable, non-radioactive method for determining consumption of solid media in *Drosophila*.

The Con-Ex approach described here was designed to assess the effects of discrete treatments on feeding (e.g. starved versus fully fed flies). Since the amount of excreted Blue 1 corresponds to the amount of consumed Blue 1, it should be possible to use Con-Ex to estimate the total volume of medium flies consume when INT, ExVial and ExMedium are all determined. Importantly, though, Con-Ex has utility even when ExVial alone is determined. The utility and ease of assessing ExVial alone raises the possibility that the method could be adapted for larger scale approaches aimed at identifying genes, genetic pathways and neural circuits that regulate feeding behavior. The possibility that Con-Ex can be used as a large scale screening platform is a significant potential advantage not shared by other current methods for assessing consumption of solid food media in *Drosophila*.

## Materials and Methods

### Research sites

All experiments were performed in the Grotewiel laboratory at Virginia Commonwealth University except those reported in Figs 4B and S6B which were performed in the Pletcher laboratory at the University of Michigan. The descriptions of materials and methods correspond to the studies in the Grotewiel laboratory except those noted in *Pletcher laboratory methods*.

### Reagents

All reagents and supplies were obtained from commercial vendors: *Drosophila* agar type II and cotton plugs for vials, Apex BioResearch Products, Genesee Scientific, San Diego, CA; saf-instant yeast, Lesaffre Yeast Corp., Milwaukee, WI; yellow corn meal (enriched degerminated), The Quaker Oats Co., Food Service Division, Chicago, IL; table sugar (sucrose), various brands, Richmond Restaurant Service, Richmond, VA; methyl 4-hydroxybenzoate, chloramphenicol, tetracycline, ampicillin and xylene cyanol, Sigma-Aldrich, St. Louis, MO; FD and C Blue No. 1, Blue No. 2, Red No. 40, Green No. 5, Red No. 4, Red No. 6 and Yellow No. 5, Spectrum Chemical Manufacturing Corp., Gardena, CA; polypropylene fly culture bottles (AS-355) and cotton plugs, Fisher Scientific; polystyrene narrow fly vials (89092-722) and capillary tubes for CAFE studies (53432-706), VWR International; feeder caps for Con-Ex studies (FCS13/16NA1), MOCAP, Park Hills, MO.

### Flies, culture media and fly husbandry

The standard control fly strain used in these studies (r[A]) was generated by backcrossing the w^+^ allele from Canton-S for 7 generations into a w^1118^ isogenic strain (Bloomington *Drosophila* Stock Center (BDSC) #5905). Lausanne-S was obtained from the BDSC (stock #4268).

Flies for all studies were reared to adulthood in culture bottles at 25 °C and 65% relative humidity under a 12-hour light/dark cycle on standard food medium consisting of 2% yeast, 10% sugar, 3.3% cornmeal, 1% agar, 2 g/L tegosept, 0.125 g/L chloramphenicol, 0.02 g/L tetracycline and 0.1 g/L ampicillin supplemented with live yeast. Flies were collected at 3–5 days of age using light CO_2_ anesthesia, separated by sex, and then placed directly in vials for analyses of media consumption. Con-Ex ExVial and INT volumes were not different when feeding studies were initiated with anesthetized versus awake flies (data not shown). All studies used age-matched flies reared, collected and tested side-by-side.

### Consumption-excretion studies overview

Agar-based food medium containing dye (dissolved in media prior to solidifying) was poured into plastic feeder caps (Fig. 1A) and allowed to cool at room temperature to solidify, placed in a humidified plastic box, and stored at 4 °C overnight. The following day, feeder caps containing media were warmed to room temperature for 1 h, inverted and placed in the open end of vials containing adult flies (Fig. 1B). The feeder caps used in these studies hold ~4.5 mL of medium (many-fold more than flies consume), have flanges that prevent them from falling into the vials and fit in the vials used so that condensation does not build-up, yet flies cannot escape. Adult flies (typically 15/vial, but see Fig. 6) in the vials consumed medium from the feeder caps (the only food source) and then excreted waste over time (Fig. 1C). A single feeder cap was used in each vial over the duration of each experiment. Feeder caps were discarded at the conclusion of feeding (Fig. 1D). The dye inside the flies (internal dye, INT) was collected via homogenization of animals in 1.5 ml of water followed by centrifugation to pellet debris. The dye excreted by flies on the walls of the vials (excreted vial dye, ExVial) was collected by addition of 3 ml of water to vials followed by vortexing (Fig. 1D). Definitions of abbreviations for all Con-Ex measures are provided in Table S1. Absorbance of the INT and ExVial dye in water extracts was determined in a spectrophotometer (Pharmacia Biotech Ultraspec 2000) (Fig. 1E) at wavelengths appropriate for each dye (Blue 1, 630 nm; xylene cyanol, 615 nm; Red 40, 504 nm; Green 5, 608 nm; Blue 2, 608 nm; Red 4, 500 nm; Red 6, 442 nm; Yellow 5, 425 nm). Absorbance values were converted to volumes of medium consumed by interpolation from standard curves of pure dyes (Fig. 1F). Extracts of flies fed medium without dye controlled for background absorbance. The standard amount of time flies were allowed to consume-excrete in the vials was 24 h, but the consumption-excretion time was varied in some studies as described. When used, starvation of flies was achieved by housing them in empty vials for 17 h. In studies that assessed mating status on Con-Ex, virgin flies were collected under brief CO_2_ and then housed at 15 flies per bottle in the presence of 15 males (mated) or in the absence of males (virgin) for 2 d prior to the initiation of Con-Ex. Flies in all studies had a single water/food source (the feeder cap) and were housed undisturbed at 25 °C and 65% relative humidity under a 12-hour light/dark cycle while consuming media from feeder caps.

### Excretion on food medium

Flies (20/vial) consumed standard medium containing 3% w/v Blue 1 from a feeder cap for 4 h (to accumulate INT dye). Flies were transferred into a new, second vial. A feeder cap containing the same medium, but no dye, was placed in the second vial and flies were allowed to excrete the previously consumed dye for 24 h (at which time all or virtually all INT dye has been excreted). The concentration of Blue 1 was increased to 3% for adequate dye signal. ExVial in the second vial was collected by adding 3 ml of water to the vials followed by vortexing. Excreted dye on the medium (ExMedium) was captured by melting the medium in each feeder cap in 25 ml of water in a microwave followed by centrifugation to pellet debris. Absorbance values of the ExVial and ExMedium dye were measured as described above. Extracts of food medium without dye were used to control for background absorbance. Absorbance values for dye collected from the vial walls and from the food medium were converted to volumes via interpolation from standard curves of pure dye dissolved in food medium. Due to low dye signal from groups with low dye concentrations (Fig. 3A), lower number of flies (Fig. S1) and after shorter periods of feeding (Fig. 4A), determining ExMedium in these studies was not possible.

### CAFE-excretion studies

Studies on CAFE-excretion of liquid medium were performed as described^41^ with modifications to capture excretion products. Adult flies (10/vial) were placed into empty food vials and prevented from escaping by foam plugs. One capillary feeding tube per vial was held in place by a plastic pipette tip placed in the foam plug such that the capillary tube extended beyond the bottom of the plug by 3–5 mm. Flies were allowed to consume-excrete liquid medium (5% sucrose and 1% Blue 1 in water) from the capillary feeding tubes for 8 hours. The original capillary tubes containing sucrose and dye were removed, the amount of liquid medium consumed from each tube was recorded, and flies were then provided with fresh capillary feeding tubes containing 5% sucrose medium without Blue 1 for 18 h. Flies were housed in the same vials while consuming medium from both the first (containing Blue 1) and second (without Blue 1) capillary tubes, thereby allowing the excreted waste from each group of animals to accumulate in the vials and on the plugs. Excreted Blue 1 on the interior surface of vials (ExVial) was collected by adding 3 ml of water to each vial followed by vortexing. Excreted Blue 1 on foam plugs (ExPlug) was collected in 3 ml of water. Absorbance of Blue 1 was measured in a UV-vis spectrophotometer at 630 nm and absorbance values were converted to volumes by interpolation from standard curves of Blue 1. Flies were either fully fed or starved for 17 h by housing them in vials with 1% agar (wet starvation) or empty vials (starvation). Starvation with either protocol led to comparable amounts of medium consumption and excretion (not shown). Flies were housed undisturbed (except for replacing capillary tubes) in an opaque polycarbonate box at 25 °C and 65% relative humidity under a 12-hour light/dark cycle throughout CAFE-excretion studies. Vials without flies were used to control for evaporation of liquid medium from capillary tubes.

### Pletcher laboratory methods

For the studies reported in Figs 4B and S6B, an equal amount of Canton-S embryos were dispensed and reared on conventional sugar, yeast, cornmeal, and agar medium (CT) to control for density of developing larvae/pupae and synchronize the emergence of adults. Newly emerged adults were placed on fresh CT diet to allow for fly mating. 8–9 day old mated female and male flies were sorted under light CO_2_ anesthetization into groups of 15 same-sex flies and transferred onto SY10 dietary medium containing 10% (w/v) sucrose, 10% (w/v) yeast, 2% (w/v) agar, 0.3% (w/v) tegosept, 0.3% (v/v) propionic acid, 0.002% (w/v) tetracycline, and 0.005% (w/v) kanamycin. Eight replicate vials were created for each sex and time point. After keeping flies on SY10 medium for three days, flies were then transferred onto SY10 diet containing 1% FD&C Blue No. 1 dispensed into the Con-Ex MoCaps for the interval specified in the figures. Time course measurement studies were started 2 hours after lights-on. All larvae/pupae/adults were maintained at 25 °C, 12-hour light/dark, and 50–60% humidity throughout the experiment. Processing of flies/vials, absorbance measurements, and data analysis were performed as described in the Grotewiel laboratory methods.

### Statistical analyses and data presentation

All statistical analyses (normality tests, unpaired two-tailed t tests, one-sample two-tailed t tests, one-way ANOVAs, Bonferroni’s multiple comparisons, Pearson correlations and linear regressions) and interpolations from standard curves were performed using Prism 6.07 (GraphPad Software Inc., San Diego, CA). Data analyzed were normally distributed and therefore parametric statistical tests were used. P values ≤ 0.05 were considered statistically significant. Data shown are mean ± S.E.M. unless noted otherwise. Power analyses were performed with StatMate (GraphPad Software Inc., San Diego, CA).

### Data availability

The datasets generated during the current study are available from the corresponding author on reasonable request.

## Electronic supplementary material

Supplementary Information
