# Supplementary material for: Measurement of solid food intake in Drosophila via consumption-excretion of a dye tracer
Source: Sci Rep. 2018 Aug 1;8:11536. doi: 10.1038/s41598-018-29813-9 (PMC6070562; doi:10.1038/s41598-018-29813-9)
Supplement: Supplementary file 1 — Supplementary Information [file 41598_2018_29813_MOESM1_ESM.pdf]

Title: Measurement of solid food intake in *Drosophila* via consumption-excretion of a dye tracer

Authors: Brandon C. Shell[1], Rebecca E. Schmitt[1,2], Kristen M. Lee[1,3], Jacob C. Johnson[4], Brian Y. Chung[4], Scott D. Pletcher[4], Mike Grotewiel[1,2,3,5,*]

Affiliations: [1]Department of Human and Molecular Genetics, Virginia Commonwealth University, Richmond, VA; [2]Human Genetics Graduate Program, Virginia Commonwealth University, Richmond, VA; [3]Neuroscience Graduate Program, Virginia Commonwealth University, Richmond, VA; [4]Department of Molecular and Integrative Physiology and Geriatrics Center, University of Michigan; [5]VCU Alcohol Research Center, Virginia Commonwealth University, Richmond, VA.

***Corresponding author:** Mike Grotewiel, Virginia Commonwealth University, Richmond, VA; email: michael.grotewiel@vcuhealth.org; ph: 804-628-4086; fax: 804-828-1124.

Shell, Figure S1

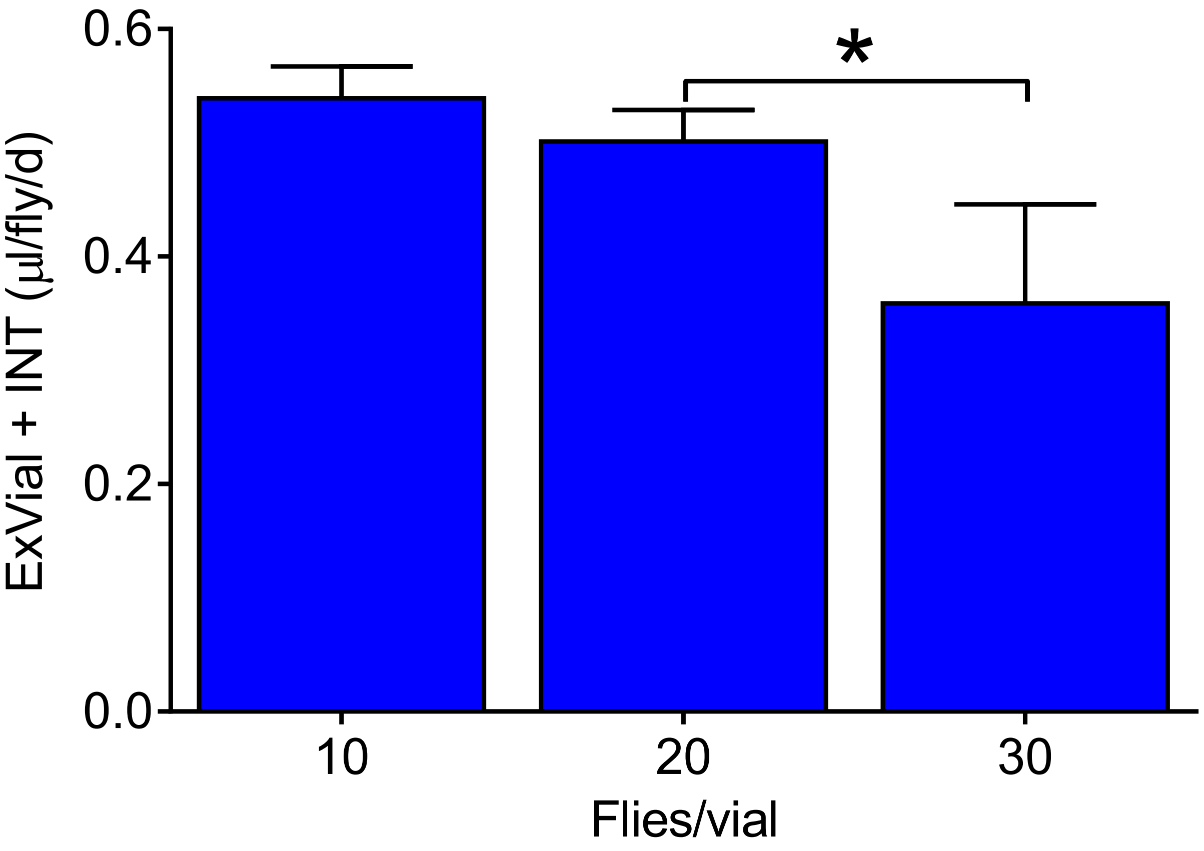

Shell, Figure S2

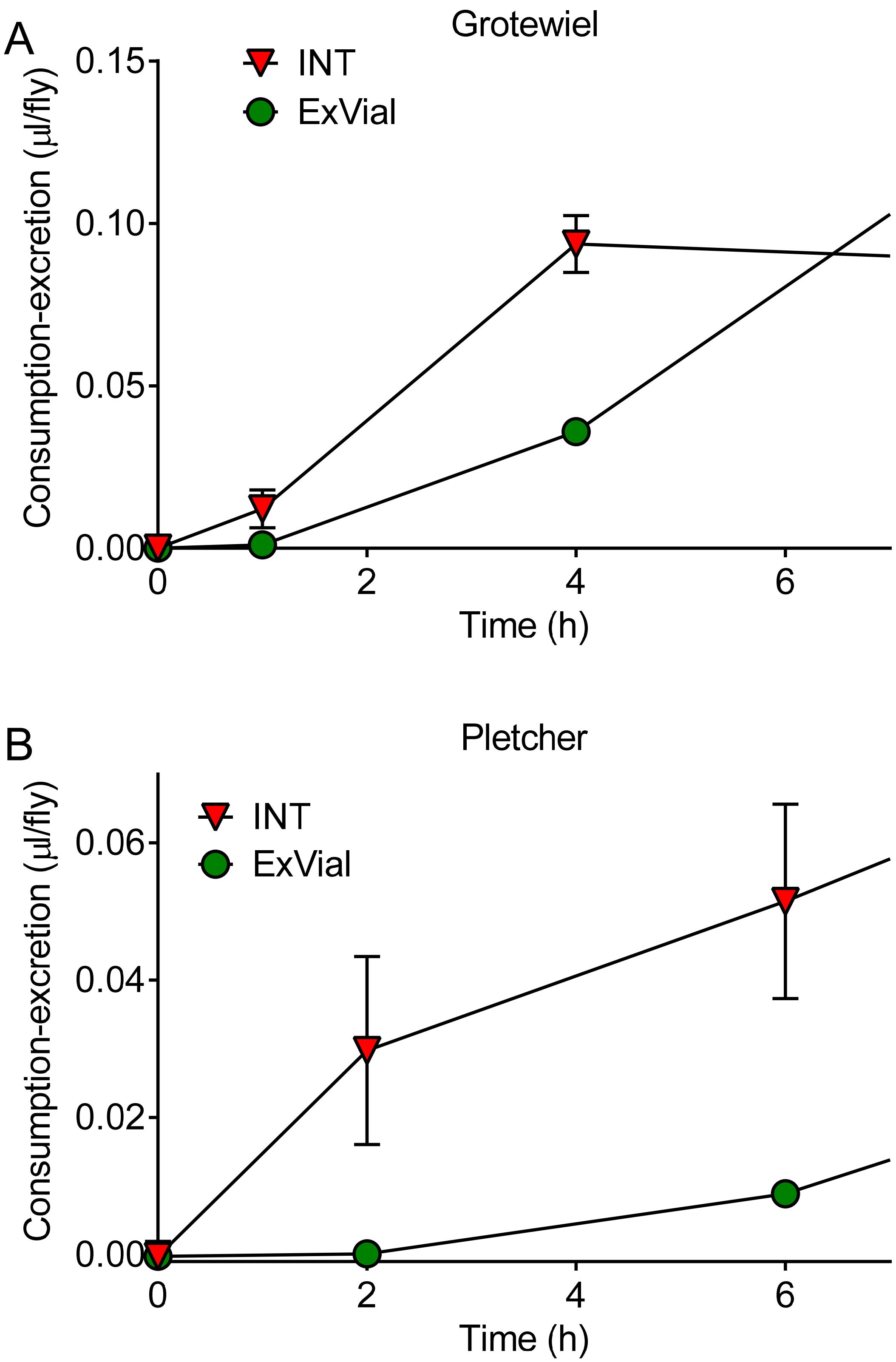

Shell, Figure S3

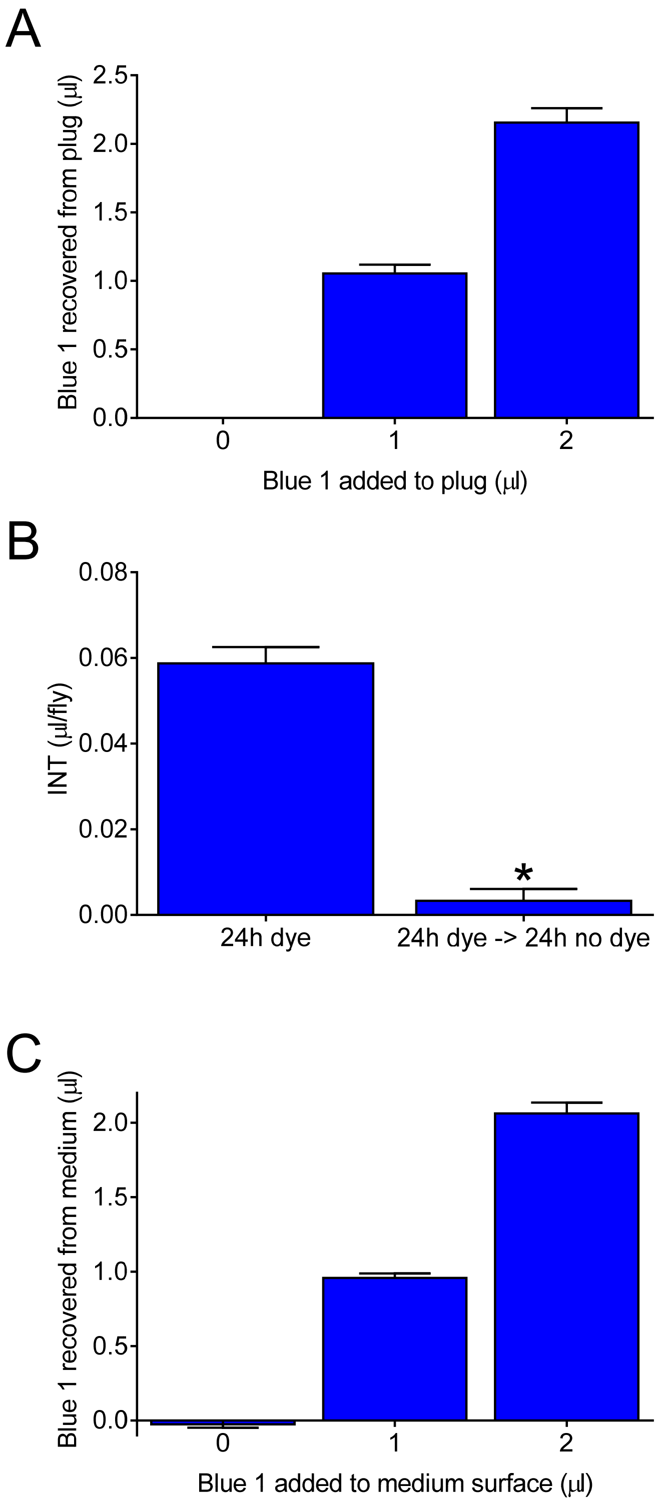

Shell, Figure S4

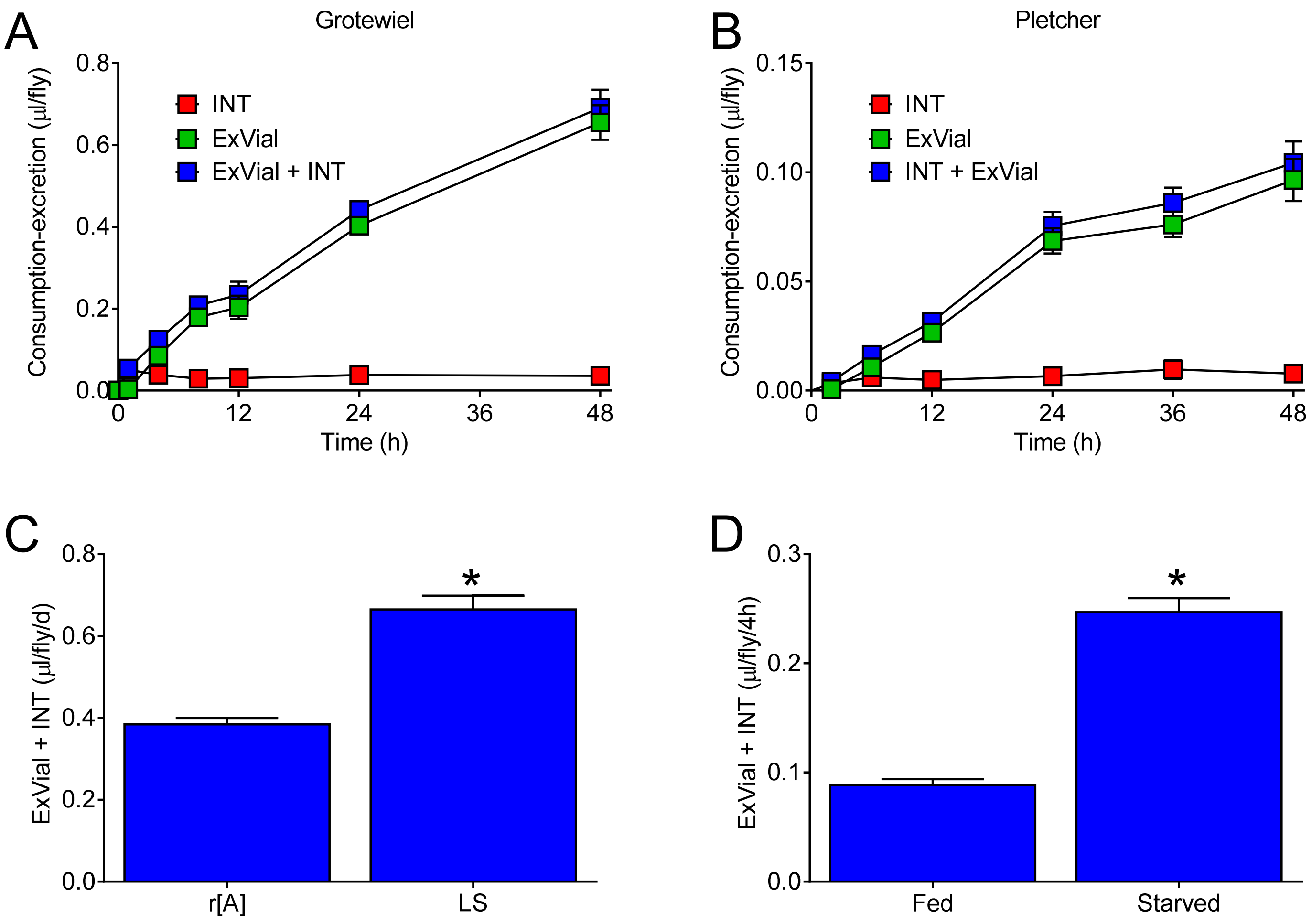

Shell, Figure S5

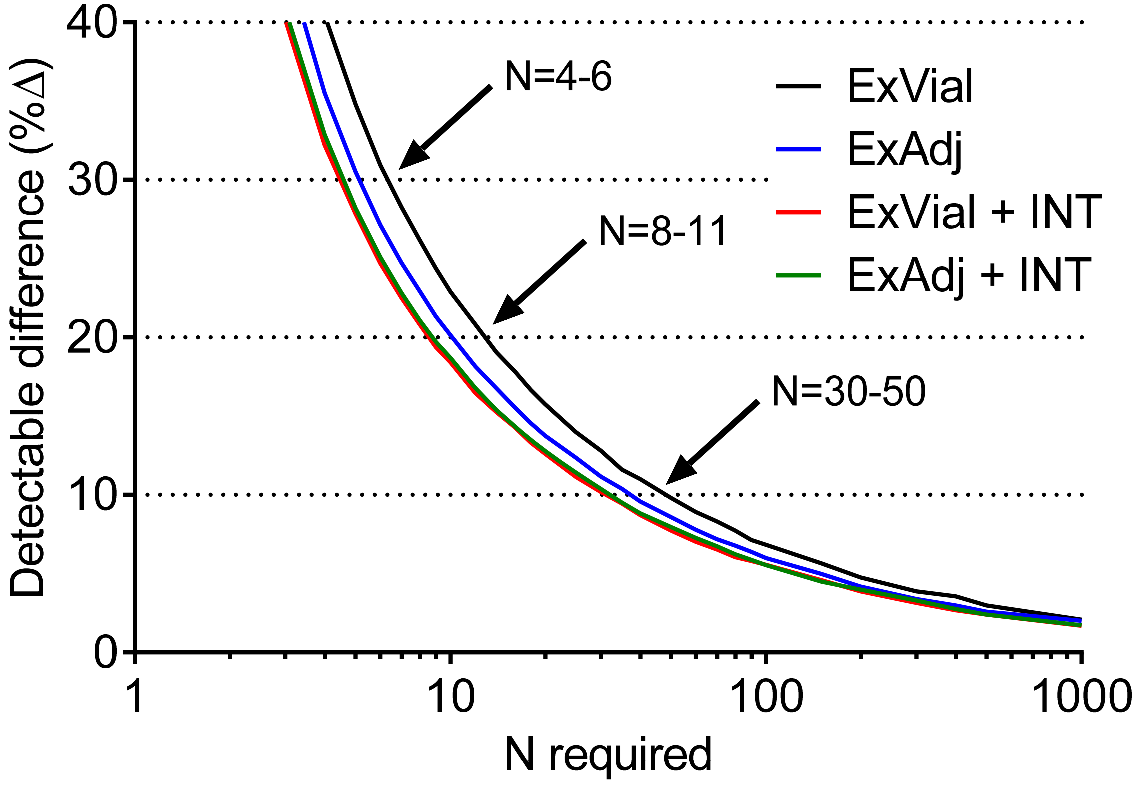

Supplementary figure legends

Figure S1. Fly density in Con-Ex. Control r[A] females at the indicated number/vial consumed 2Y10S3C with 1% Blue 1 for 24 h while excreting waste. The density of flies significantly influenced ExVial+INT (one-way ANOVA, p<0.0001, n=16). ExVial+INT was lower in flies housed in groups of 30/vial than other groups (*Bonferroni's multiple comparison test, p=0.0010 to <0.0001), but was not different in groups of 10 and 20 flies (Bonferroni's multiple comparison, p=0.9255).

Figure S2. Early time-points of INT and ExVial. Detailed view of early time-points from the data in Fig. 4.

Figure S3. Methodological background for measuring excretion on foam plugs and food medium. (A) The amount of Blue 1 recovered from foam plugs were indistinguishable from the volume added (individual one sample t tests, p=0.2829 to 0.9999, n=3). (B) INT in r[A] females that had consumed dyed food for 24 h (24 h dye) was significantly greater than in flies after subsequently consuming media without dye for an additional 24 h (24 h dye -> 24 h no dye) (*two-tailed t test, p<0.0001, n=12). INT remaining in 24 h dye flies was significantly greater than zero (one sample two-tailed t test, p<0.0001), whereas INT remaining in 24 h dye -> 24 h no dye flies was not distinguishable from zero (one sample two-tailed t test, p=0.2518). (C) The volume of Blue 1 extracted from the food medium in feeder caps was indistinguishable from the volumes added to the surface of the medium (individual one sample t tests, p=0.2607 to 0.4577, n=4).

Figure S4. Targeted Con-Ex studies in males. (A and B) Time-dependent accumulation of INT, ExVial and ExVial+INT in r[A] (A, Grotewiel laboratory) and Canton-S (B, Pletcher

laboratory). ExVial and ExVial+INT increased in a linear fashion with time ($p<0.0001$, $R^2=0.8450-0.9108$, n=7-8 per time-point). (C) ExVial+INT was greater in LS compared to r[A] males (two-tailed t test, $p<0.0001$, n=8). (D) Starvation increased ExVial+INT in r[A] males (two-tailed t test, $p<0.0001$, n=8).

Figure S5. Power analysis of Con-Ex data. Data are the N required (number of replicates in each of two groups; X-axis) to detect the indicated % changes between the means of the two groups (Y-axis) using an unpaired two-tailed t test with 80% power when measuring ExVial, ExAdj, ExVial+INT and ExAdj+INT based on average standard deviations for the four measures of 0.058, 0.076, 0.057 and 0.081 µl/fly/d, respectively. The number of replicates required to detect 30%, 20% and 10% differences between means are indicated by arrows.

Measure	Definition
INT	The volume of internal dye
ExVial	The volume of dye excreted on the vial walls
ExMedium	The volume of dye excreted on the food medium
ExAdj	The total volume of dye excreted calculated as the ExVial volume adjusted for the ExMedium volume
ExVial+INT	The volume of internal dye plus the volume of dye excreted on the vial walls
ExAdj+INT	The total amount of dye consumed-excreted calculated as the internal volume of dye plus the volume of dye excreted in the vial adjusted for the volume excreted on the food medium
ExPlug	The volume of dye excreted on the foam plug

Table S1. Definitions of measures used in Con-Ex studies. See main text for details.

	Media						
	2Y10S3C	15Y10S3C	30Y10S3C	2Y20S3C	2Y30S3C	2Y40S3C	2Y10S10C
**Yeast** (g/L)	20	150	300	20	20	20	20
Protein (g/L)	10	75	150	10	10	10	10
Fiber (g/L)	5.4	40.5	81	5.4	5.4	5.4	5.4
Other Carbs (g/L)	1.2	9	18	1.2	1.2	1.2	1.2
Fats (g/L)	1.2	9	18	1.2	1.2	1.2	1.2
kCal/L	78	585	1170	78	78	78	78
**Sugar** (g/L)	100	100	100	200	300	400	100
Protein (g/L)	0	0	0	0	0	0	0
Fiber (g/L)	0	0	0	0	0	0	0
Other Carbs (g/L)	100	100	100	200	300	400	100
Fats (g/L)	0	0	0	0	0	0	0
kCal/L	375	375	375	750	1125	1500	375
**Cornmeal** (g/L)	33	33	33	33	33	33	100
Protein (g/L)	2.4	2.4	2.4	2.4	2.4	2.4	7.3
Fiber (g/L)	2.4	2.4	2.4	2.4	2.4	2.4	7.3
Other Carbs (g/L)	23.2	23.2	23.2	23.2	23.2	23.2	70.3
Fats (g/L)	0.6	0.6	0.6	0.6	0.6	0.6	18.2
kCal/L	110	110	110	110	110	110	333
**Total**							
Protein (µg/µl)	12.4	77.4	152.4	12.4	12.4	12.4	17.3
Fiber (µg/µl)	7.8	42.9	83.4	7.8	7.8	7.8	12.7
Other Carbs (µg/µl)	124.4	132.2	141.2	224.4	324.4	424.4	171.5
Fats (µg/µl)	1.8	9.6	18.6	1.8	1.8	1.8	19.4

Table S2. Components and calories in food media. Values that changed relative to 2Y10S3C (standard) medium are in red.

Figure	Statistical test	ExVial	ExAdj	ExVial+INT	ExAdj+INT
3A	One-way ANOVA: Blue 1 concentration	0.3674	0.3681	0.2255	0.3056
	Bonferroni's: 0.25 vs 0.5%	0.6216 (-7.7%)	0.6262 (-7.7%)	0.5707 (-8.1%)	0.5423 (-8.0%)
	Bonferroni's: 0.5 vs 1.0%	0.2778 (11.2%)	0.2771 (11.2%)	>0.9999 (4.6%)	0.9584 (6.5%)
	Bonferroni's: 1.0 vs 2.0%	>0.9999 (-6.0%)	>0.9999 (-5.9%)	0.4174 (-12.6%)	0.5550 (-10.8%)
S1	One-way ANOVA: density	<0.0001	<0.0001	<0.0001	<0.0001
	Bonferroni's: 10 vs 20	0.5045 (-7.5%)	0.5059 (-7.5%)	0.6170 (-7.0%)	0.5800 (-7.2%)
	Bonferroni's: 20 vs 30	0.0003 (-29.9%)	0.0003 (-29.1%)	0.0007 (-28.4%)	0.0005 (-28.6%)
4A	One-way ANOVA: time	<0.0001	<0.0001	<0.0001	<0.0001
	Bonferroni's: 0 vs 1 h	>0.9999 (n/a)	<0.9999 (n/a)	>0.9999 (n/a)	>0.9999 (n/a)
	Bonferroni's: 1 vs 4 h	0.7516 (3488%)	0.7555 (2787%)	0.0003 (886%)	0.0043 (955%)
	Bonferroni's: 4 vs 8 h	0.0015 (248%)	0.0015 (247%)	0.0135 (65.2%)	0.0063 (87.1%)
	Bonferroni's: 8 vs 12 h	0.2749 (36.7%)	0.2767 (36.6%)	0.5209 (21.3%)	0.4153 (24.7%)
	Bonferroni's: 12 vs 24 h	<0.0001 (88.0%)	<0.0001 (88.2%)	<0.0001 (57.3%)	<0.0001 (65.0%)
	Bonferroni's: 24 vs 48 h	<0.0001 (44.8%)	<0.0001 (44.7%)	<0.0001 (32.2%)	<0.0001 (35.8%)
5A	t test: r[A] vs LS	<0.0001 (99.9%)	<0.0001 (94.3%)	<0.0001 (92.3%)	<0.0001 (90.1%)
6A	t test: media dilution	<0.0001 (50.4%)	<0.0001 (42.4%)	<0.0001 (52.4%)	<0.0001 (44.5%)
6B	t test: yeast supplementation	<0.0001 (-65.8%)	<0.0001 (-58.7)	<0.0001 (-56.7%)	<0.0001 (-53.3%)
6C	t test: sucrose supplementation	0.0001 (-38.9%)	<0.0001 (-51.6%)	0.0003 (-32.2%)	<0.0001 (-45.4%)
6D	t test: cornmeal supplementation	0.7022 (-4.8%)	0.0411 (-26.4%)	0.2532 (-10.7%)	0.0121 (-27.0%)

Table S3. Statistical reanalysis of individual measures from Con-Ex studies. Columns indicate the corresponding figure, the statistical tests used and the measures analyzed (ExVial, ExAdj, ExVial+INT, ExAdj+INT). Data are the p values for the indicated statistical tests and (for tests involving two groups) the percent changes between groups in parentheses. Statistically significant p values (and percent changes) are indicated in red. The percent changes in ExVial, ExAdj, ExVial+INT and ExAdj+INT correlated at r = 0.993 to 1.000 (Pearson correlation matrix, p = 2.1E-13 to 1.35E-22) for all cells and at r =0.792 to 0.999 (Pearson corrlation matrix, p = 0.0191 to 1.05E-11) for cells with significant changes.
